# Pharmacist joint-working with general practices: evaluating the Sheffield Primary Care Pharmacy Programme. A mixed-methods study

**DOI:** 10.3399/bjgpopen18X101611

**Published:** 2018-10-17

**Authors:** Iuri Marques, Nicola Jane Gray, Jo Tsoneva, Peter Magirr, Alison Blenkinsopp

**Affiliations:** 1 Senior Research Fellow in Safe Use of Medicines, School of Pharmacy and Medical Sciences, University of Bradford, Bradford, UK; 2 Independent Pharmacist Researcher, Green Line Consulting Limited, Manchester, UK; 3 Pharmacy Development Manager, NHS Sheffield Clinical Commissioning Group, Sheffield, UK; 4 Quality and Strategy Lead for Medicines Management, NHS Sheffield Clinical Commissioning Group, Sheffield, UK; 5 Board Member, Wicker Pharmacy, Sheffield, UK; 6 Professor of the Practice of Pharmacy, School of Pharmacy and Medical Sciences, University of Bradford, Bradford, UK

**Keywords:** primary health care, general practice, community pharmacy services, patient care

## Abstract

**Background:**

The NHS in the UK supports pharmacists’ deployment into general practices. This article reports on the implementation and impact of the Primary Care Pharmacy Programme (PCPP). The programme is a care delivery model that was undertaken at scale across a city in which community pharmacists (CPs) were matched with general practices and performed clinical duties for one half-day per week.

**Aim:**

To investigate (a) challenges of integration of CPs in general practices, and (b) the perceived impact on care delivery and community pharmacy practice.

**Design & setting:**

This mixed-methods study was conducted with CPs, community pharmacy employers (CPEs), scheme commissioners (SCs), and patients in Sheffield.

**Method:**

Semi-structured interviews (*n* = 22) took place with CPs (*n* = 12), CPEs (*n* = 2), SCs (*n* = 3), and patients (*n* = 5). A cross-sectional survey of PCPP pharmacists (*n* = 47, 66%) was also used. A descriptive analysis of patient feedback forms was undertaken and a database of pharmacist activities was created.

**Results:**

Eighty-six of 88 practices deployed a pharmacist. Although community pharmacy contracting and backfill arrangements were sometimes complicated, timely deployment was achieved. Development of closer relationships appeared to facilitate extension of initially agreed roles, including transition from ‘backroom’ to patient-facing clinical work. CPs gained understanding of GP processes and patients’ primary care pathway, allowing them to follow up work at the community pharmacy in a more timely way, positively impacting on patients' and healthcare professionals’ perceived delivery of care.

**Conclusion:**

The PCPP scheme was the first of its kind to achieve almost universal uptake by GPs throughout a large city. The study findings reveal the potential for CP–GP joint-working in increasing perceived positive care delivery and reducing fragmented care, and can inform future implementation at scale and at practice level.

## How this fits in

Previous studies in other countries have shown that pharmacists working in general practices can enhance patient care and enable better management of GP workload involving medicines and prescriptions. Large scale deployment of CPs to general practices has not been reported in the literature. In the present study, CPs were able to substitute for medicines-related GP roles, including post-discharge medicines reconciliation, medication reviews, and prescription queries. Working in general practices allowed CPs to gain understanding of the patient's complete pathway in primary care; access to patient records, and the relationships established between CPs and the practice team, allowed CPs to provide more effective patient care in community pharmacy.

## Introduction

General practices in the UK are experiencing increasing pressures from workload, an ageing population, and difficulty in recruiting and retaining GPs.^1–3^ Integration of pharmacists in general practice has been suggested as one solution to easing these pressures.^3–8^

Since 2015, NHS England has been recruiting practice-based pharmacists, with a target of 20% of practices by 2020–2021.^9–11^ The Prime Minister’s Challenge Fund provided over £100 million for 2015–2016 to increase access.^12^ One UK city, Sheffield, decided to deploy local CPs in all general practices; a programme known as the PCPP.^13^ The aim was to redistribute GPs’ workload and enhance patient care over a 12-month period by providing support with (a) repeat prescriptions, (b) medicines reviews, (c) liaison with community pharmacies, (d) support to patients with long-term conditions on complex medicines regimens, and (e) support to care home patients.^13^

Previous studies and reports have identified practice pharmacists’ contributions to medicines reviews and monitoring,^6–8,14,15^ error minimisation,^5,16^ treatment of minor ailments,^3^ multimorbidities, long-term conditions,^17^ and reduction of medicines waste.^7^ Pharmacists qualified as prescribers have also contributed to diagnosis and prescribing.^14,15^ Pharmacists can also reduce fragmented care by improving communication between GPs and community pharmacy.^8^ However, most studies were conducted outside the UK (notably Australia, Canada, and New Zealand) and did not focus on CPs. The PCPP was the first to integrate CPs in general practice at scale, and there are no published studies from the UK or elsewhere investigating how CP deployment in general practices can contribute to general practice work.

GP perspectives of the PCPP were the subject of a separate study.^18^ This article complements previous work by reporting findings from the community pharmacy perspective, aiming to investigate how the GP–CP collaboration can be strengthened in the future by focusing on (a) challenges of integration of CPs in general practices (scheme implementation), and (b) perceived impact on the delivery of care and community pharmacy practice.

## Method

Mixed methods of data collection comprised (a) semi-structured interviews with CPs, CPEs, and SCs﻿, (b) cross-sectional survey of CPs, (c) patient feedback forms, and (d) secondary analysis of service provision data.

Interview schedules were developed and reviewed by the research team and two patient reference group members. Data saturation and sample sizes were considered in planning, based on guidance from the literature^19^ and previous experience conducting similar studies: for CPs and patients it was decided that there would be 8–12 interviews, and for CPEs representation from two local chains, two national multiples, and two independents (*n* = 6) were deemed sufficient. All SCs (*n* = 3) were invited. For CPEs, since the majority of pharmacies participating were local chains or large multiples, all CPEs were invited to maximise the response rate. For maximum reach, interview findings were triangulated with patient feedback forms, and survey and service provision data.

SCs posted interview invitation letters, participant information sheets, and consent forms to 24 CPs and all CPEs. Purposive sampling^19 ^was used for a maximum diversity sample: CPs were stratified by sex, practice size (from 3042–29 390 patients), pharmacy type, proximity to general practice, and training versus non-training practices. Invitations were sent to all CPEs. SCs randomly selected two PCPP practices to invite 30 PCPP patients (*n *= 15 each) by post if they had had a face-to-face appointment with their CP. SCs were sent the interview invitation letters, patient information sheets, and consent forms. Reminders were sent 15 days after initial contact. With the exception of SCs, personal details of participants were only made known to the research team by those who returned the signed consent form, with lists of CPs and CPEs held by SCs and lists of patients held by general practices.

Face-to-face or telephone interviews with CPs, CPEs, and SCs were conducted by the research team. Patient telephone interviews were undertaken by two patient reference group members who were trained in conducting interviews. Interviews were audio-recorded and transcribed verbatim, expect for patient interviews, where written notes were taken.

CP interview transcripts were used with published literature to develop a questionnaire to capture and quantify CPs’ attitudes and experiences. Likert scales ranging from 1 (strongly disagree) to 5 (strongly agree) were used to capture CPs’ level of agreement with statements drafted. Closed questions about work settings and demographics, and two open questions about perceived impact of CPs’ work on patient safety and community pharmacy practice, were included.

The draft questionnaire was validated through six cognitive interviews with experienced practice pharmacists who were also asked to complete it and discuss whether it was clear, unambiguous, and manageable. The validated questionnaire was posted to all CPs (*n* = 73) with one reminder sent after 15 days.

An anonymised patient feedback form was developed by a patient reference group, which included a question to capture patients’ experiences of seeing a CP: 'How do you think you have benefitted from seeing the pharmacist today?' This form was provided to patients by the practice and collected by the SCs.

Anonymised aggregated service provision from CP work were analysed. Activities logged by CPs were tasks that could be delegated to CPs and agreed by GPs and CPs prior to the scheme. These included agreed estimates of the time usually taken by GPs to complete them. This allowed an estimate of how much GP time CPs covered.

Interview and survey data were collected during April 2017–August 2017. Patient feedback forms and service provision data were collected during October 2015–June 2016. Data were analysed following the theoretical framework of Glasgow *et al* ^20^ and Proctor *et al*.^21^ This involved the identification and refining of emerging codes and themes, organised into an analysis grid.^22^ Data analysis was then reviewed and consensus reached regarding codes and themes. Descriptive analysis of service provision data and the questionnaire were conducted using SPSS (version 22).

## Results

### Study participants

Interviews were conducted with CPs (*n* = 12), CPEs (*n* = 2, one representing a local chain, and one an independent pharmacy), SCs (*n* = 3), and patients (*n* = 5), as shown in Table 1, with data saturation reached for CPs and SCs (the latter being 100% of the population). Feedback forms were completed by 58 patients. Of all CPs in the PCPP scheme (*n* = 73), 47 completed and returned it (64% response rate). Regarding service provision data, a total of 18 044 individual activities were recorded by CPs between October 2015 and June 2016.

**Table 1.** Interview participants and response rates

Participants	Invitations sent, *n*	Agreed to participate, *n*	Response rate, %
Community pharmacists	24	12	50
Community pharmacy employers	6	2	33
Scheme commissioners	3	3	100
Patients	30	5	17

### Scheme set-up, challenges to implementation, and solutions found

In order to implement the scheme, SCs engaged with the 88 general practices in the city and written communication was sent inviting them to opt into 'wider extended access' initiatives, of which one strand was to work with a CP. Simultaneously, CPEs and CP contractors were invited to a launch event and then all 126 community pharmacies in the city were contacted to invite CPs to be involved. A mixed model of engagement was applied, whereby commissioners liaised with CPs who wished to be involved and their employers to negotiate inclusion in the PCPP scheme. As a result, 86 of the 88 general practices (98%) in the city had a pharmacist join their team for some or the entirety of the scheme. This study and data collected focused on CPs and patients only, and their contribution to the PCPP scheme.

#### Recruitment and release of CPs

**Figure 1.**
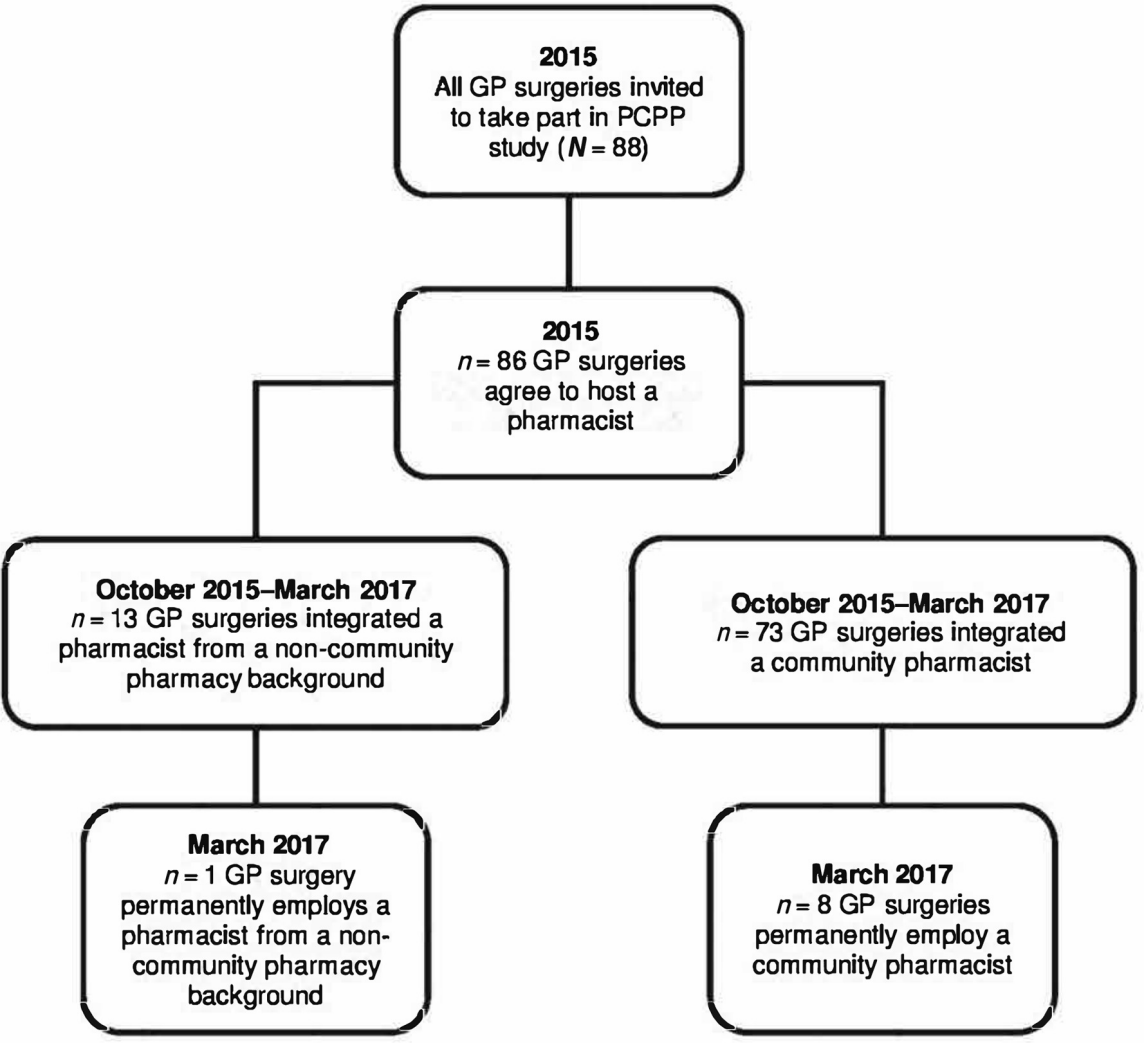
Flowchart of PCPP scheme.

Recruiting CPs proved challenging. Given the high uptake of general practices willing to participate, a shortfall of CPs meant that invitations were sent to a small number of pharmacists from non-community backgrounds, for example, those employed by clinical commissioning groups or primary care pharmacists. Seventy-three of 86 PCPP pharmacists were CPs (Figure 1); some of these agreed to work at more than one practice (17% of survey responders).

Initially, the plan was for CPs to work at the general practices for one half-day per week. Although survey data showed that this happened for the majority of cases (57%), the time pharmacists spent at the practice varied depending on the CP's ability to provide cover (including the willingness of locums to work for only half a day). For example, seven CPs surveyed reported working for one day every fortnight, meaning general practices experienced longer periods of time between sessions. Two geographically close pharmacies shared a locum to cover for the two CPs' work at two different practices, and another pharmacy released their CP for one half-day but booked a locum for a full day, to enable the CP to focus on paperwork or other activities at the pharmacy in the afternoon. Notwithstanding the solutions to release CPs to work in general practice, one CPE reported that this was not lucrative, and was therefore a barrier to the uptake of future schemes (Box 1).

**Box 1.** Scheme set-up, challenges to implementation and solutions found

**Recruitment and release of CPs** *'It just happened to be that in a couple of cases we had pharmacists who were happy to do some additional half-days when they were not down to be working. We had some people who were happy to cover for half a day and who were not looking for full days.'* (CPE3, interview) **Training** *'Nobody taught us to use SystmOne. It was just you turned up on that first morning. Your SMART card had the access put on it, you were shoved in a room by yourself, "get on with it".'* (CP1, interview) **GP knowledge of CPs’ clinical contribution to care** *'I don’t think they knew what to do with me so they probably need somebody to explain to them.'* (CP15, interview) *'Once you get to the point of trust, then you start to see “oh, so you could do that for me” and “you’re interested in doing that?” So you start to see that sort of innovation start to occur at a practice level.'* (SC2, interview)

#### Training

Formal training was not provided but CPs were given general resources from the Pharmaceutical Services Negotiating committee, and specific resources, such as a menu of GP clinical system settings. In the survey, 36% of responders agreed that they had not received adequate training, which was a sentiment echoed by CPs interviewed, who were initially unsure what work they were meant to do while in the practice. This posed a challenge to developing closer collaborations and relationships (Box 1).

#### GP knowledge of CPs’ clinical contribution to care

Some CPs reported that practice teams did not know how to use their skills, linking this to the lack of knowledge of what CPs can do, and felt practice teams would have benefited from training in advance (Box 1). This lack of knowledge about CPs’ skills led some CPs to believe that practice teams felt that they would add nothing of value, hindering the successful implementation of the scheme. One SC pinned this challenge on the lack of trust by the practice team in how CPs could contribute to their work and the traditionally perceived overlap between roles (Box 1). Effective joint-working was reported by CPs to be effective when their medicines-related knowledge and skills were perceived as valued, and their contribution recognised as an important component to enhanced care delivery, which is a feature of successful collaborations.

### Perceived impact of GP–CP joint-working on care delivery

#### GP work delegated to CPs

One aspect of the PCPP scheme was the amount of work and tasks that were usually done by GPs and that were delegated to CPs. The high number of activities reported is sufficient to extrapolate estimates of GP times covered by CPs during the PCPP. The 18 044 tasks recorded in the PharmOutcomes database represented a total estimate of 3171.2 GP hours that would otherwise have been spent on medicines-related tasks and were released by CPs (Table 2).

**Table 2.** Service provision data: estimated GP time covered by CPs

Services provided	Frequency, *n*	Estimated time covered by CPs (hours)
Seeing patients face-to-face at the GP surgery	760	231.8
Seeing patients in their own home	51	28.0
Telephone contacts with patients	819	201.3
Case note reviews	11 119	1835.6
Activities with no recorded ‘setting’	5295	874.5
Total recorded interventions	18 044	3171.2

The most frequently reported work by CPs in the survey was dealing with prescription queries from other CPs or patients about new medicines or changes in medicines (94%), followed by post-discharge medicines reconciliation (89%), and medication reviews (83%). Although it was not an expectation that CPs would do patient-facing work, face-to-face medication reviews were recorded in 41% of practices, and domiciliary medicines reviews in 23%. Findings indicate a high level of autonomous working by CPs, with only one in 10 tasks resulting in a referral to GPs or practice teams. Post-discharge medicines reconciliation was a time-critical activity, which was sometimes undertaken by GPs and sometimes delegated to administrative staff, and CPs reported that GPs particularly valued this work being done by them (Box 2).

**Box 2.** Perceived impact of GP–CP joint-working on the delivery of care

**GP work delegated to CPs** *'The hospital has added something or they’ve taken something off … and the receptionists were getting spooked by it* […] *So when I came along, this is somebody who knows about the medicines and what looks like likely and unlikely combinations* […] *So there was … just this gap, this vacuum waiting to be filled.'* (CP2, interview) **Delivery of enhanced care** *'It’s reduced their sort of paperwork time significantly* […] *potentially you could say that that does mean that they could maybe have more appointments and so on.'* (SC1, interview) *'I think it's a good idea* [to be seen by a CP] *as normally I would have to wait 6 weeks to see a doctor. Also I felt he had more time to discuss things about my meds.'* (Patient feedback form, comment 4) **Patient outcomes and patient safety** '[The practice] *hadn’t got recalls on people taking methotrexate,* [patients] *weren’t having their blood tests. People on* [new oral anticoagulants] *weren’t having their blood test* [to check] *kidney function* [...] *We picked up a few old ladies who’d got really low kidney function.'* (CP1, interview) *'Three patients were identified that were taking regular OTC meds —﻿ 2 NSAIDs, 1 aspirin 25 mg — which were either contraindicated, inappropriate, interacted with, or in combination with current meds were contributing to/exacerbating conditions the patient had previously reported.'* (CP16, survey) *'The pharmacist service has benefited me by taking less tablets per day. I know it takes time to get it correct but the service has been first class. Also the* [reduction in] *swelling in my ankles has been a big plus. Thank you once again*.' (Patient feedback form, comment 2)

#### Delivery of enhanced care

Both CPs and SCs felt that having CPs working in general practice had improved the way in which care is delivered to patients. Having tasks delegated to CPs meant that GPs’ workload was optimised and they could focus on other priorities. CP input was reported to be particularly welcomed when tasks were perceived by GPs as potentially complex, but CPs regarded them as within their repertoire of routine medicines-related work (Box 2).

Patient feedback forms revealed that patients felt that care received and their experience had been enhanced by seeing their CP (Box 2). They were positive about the scheme and its impact on their care. For example, one patient reported feeling that being seen by a CP decreased waiting times to be seen at the general practice, while other patients felt that CPs have better knowledge of medicines and that their appointments with CPs resulted in an improved understanding of their medicines, compared with when they had these appointments with GPs. Similarly, a number of patients reported that appointments with their CP were more detailed, with one patient reporting that the very first thorough review of medicines occurred as a consequence of seeing a CP as opposed to a GP (Box 2). As a result, 49 of the 59 patients (83%) who completed the feedback form were 'extremely likely' to recommend this service, and nine (15%) 'likely' to do so.

Survey responders agreed that their work minimised medicines waste (60%) and enhanced care delivery (89%), with interview data providing detailed accounts of work around medicines synchronisation, optimisation, and reconciliation, outcomes of which were valued by patients.

#### Patient outcomes and patient safety

Survey responders gave examples of instances where they perceived to have contributed to increased safety by identifying and correcting errors previously undetected, and preventing avoidable harm, including required follow-ups or blood tests; patients on wrong or discontinued medicines; and problematic prescribing (for example patients on multiple painkillers). Survey findings showed a large proportion of CPs agreeing that their work improved patient safety (85%). Perceived improved safety was further explored in the interviews, where CPs had examples of instances where their work identified and rectified potential errors, which would have otherwise remained undetected (Box 2).

### Perceived impact on community pharmacy practice of relationships established between GPs and CPs during the PCPP

#### Improved GP–CP relationships

The majority of survey responders reported that their relationship with the practice team had improved after the PCPP scheme (83%), and that communication between community pharmacy and general practice was more effective as a consequence (78%). Moreover, one pharmacist perceived extra referrals to community pharmacy as the consequence of GPs gaining understanding of the CP's role. Box 3 presents survey pharmacists' reported impact on their community pharmacy practice, which included, for example, greater understanding of the general practice work, more respect from GPs, the practice team and patients, more referrals to pharmacy, and quicker access to the practice team.

**Box 3.** Perceived impact of relationships established between GPs and CPs during the PCPP on community pharmacy practice

**Improved GP–CP relationships** *'Before* [the scheme], *if I’ve got a query about medication for patients* [I would] *often leave it with the receptionist … but on occasions I felt I needed to ask the question twice because … the thing that I was asking hadn’t been transferred in the way that I wanted, so I got an answer to a slightly different question. Whereas once I started to work there, I think the reception staff felt a little bit more like I belonged with them as well, so … if I went late morning, they’ll say “They’re free —﻿ just go through”.'* (CP13, interview) *'I think I’ve certainly had a few more of the* [New Medicines Service referrals by a GP] *because* [it is] *an opportunity to speak to the GPs and fill in their gaps on knowledge.'* (CP14, interview) **Improved care in community pharmacy** *'The pharmacy customers felt reassured (and were more confident in asking advice) at the pharmacy after seeing me at the surgery;* [GP–CP joint-working] *made the process of reordering/chasing up prescriptions smoother and easier. Working together with the staff at the surgery has been useful to sort out of stock/prescription issues in the best interest of the patient.'* (CP3, survey) *'I think a lot of things I can see there in the surgery then send it back to the pharmacy in a very quick way.'* (CP15, interview)

#### Improved care in community pharmacy

Interview and survey participants agreed that CPs’ integration in general practices contributed to more effective care delivery at the pharmacy, with pharmacists reporting identifying medicine issues at the practice for follow-up at the pharmacy. CPs also reported that working at the practice expedited the delivery of care at the pharmacy. This was partly owing to their access to patients’ healthcare records, which were unavailable in community pharmacy. However, not all general practices were open to allowing CPs to access patients’ records (Box 3).

## Discussion

### Summary

Pharmacists were deployed in 98% of the city’s general practices, the majority of which were matched with a CP. Most CPs appear to have become integrated into the practice work, covering specific parts of GPs’ medicines-related work, with 3200 hours of GP time substituted by CPs. The work covered included reconciling complex polypharmacy changes for patients discharged from hospital; conducting medication reviews; and supporting administrative staff dealing with medicines queries to make repeat prescription processing more efficient. Study findings show that CPs contributed to detecting medicines-related problems and avoiding preventable patient harm. Most patients found engagement with CPs informative and helpful, although practice teams could have better communicated the inclusion of the new pharmacist. Not all CPs were successfully integrated, and inter-practice variation in deployment of CPs in patient-facing clinical work appears to have been affected by (a) the level of trust developed between key practice staff and CPs, (b) the practice teams’ understanding of the range of roles and tasks that could be allocated to CPs and their perceptions of CP capability, and (c) CPs’ proactivity and professional self-esteem. CPs whose work was local to the practice were able to provide a more seamless service for patients and complete episodes of care for medicines-related problems.

The relationships and trust developed during the PCPP appeared to have continued after the scheme ended (nine general practices subsequently employed a pharmacist on a permanent basis, of which eight were CPs, and other CPs continue to be invited in by practice teams to do work because they recognise the clinical benefit and value CPs’ contribution).

### Strengths and limitations

The use of mixed-methods with data from multiple sources was a strength, providing a broad picture of the CP–GP joint-working programme over time. Purposive recruitment maximised inclusion of a wide range of experiences. While a published report separate to this study has reported the views of GPs,^18^ this is the first study to investigate the CP perspective.

The small number of CPEs interviewed meant that data saturation may not have been reached. Nevertheless, findings provide insight into challenges faced and solutions found to release CPs to work in general practices. SCs enhanced this picture because of their experience of working with CPEs in the scheme set-up. Only five patients agreed to be interviewed, and a small number completed the feedback form. It is possible that patients with less positive experiences were less likely to take part. Triangulating findings from patient interviews and patient feedback forms strengthened the analysis. These efforts notwithstanding, interviewing more patients and CPEs could have provided a more complete picture.

### Comparison with existing literature

Pharmacists have long been recognised as valuable members of practice teams.^5–7,14–17,23,24^ However, although several countries have introduced pharmacists into general practice, this so far has often been restricted to small localities and the pharmacists deployed were not from a community background. A clinical trial in England reported that deployment of pharmacists in general practice was associated with significant reduction of medication errors compared with general practices without pharmacist support, although the background of the pharmacists involved was not clear.^25^ A 2008 Canadian study of integration of pharmacists with previous experience in community pharmacy also reported similar benefits.^26^ However, only seven pharmacists were deployed, and they no longer worked in community pharmacy, precluding the opportunity to investigate how CPs' skills specifically can contribute to practice work, or how CP–GP joint-working can be strengthened to minimise fragmented care. The PCPP was, to the authors' knowledge, the first scheme integrating CPs at scale.

One of the conditions of engagement in the primary care access scheme was that every participating practice had to deploy a pharmacist. While this was accomplished in 98% of practices, mixed levels of practice engagement were demonstrated by service provision data, which showed a range from hundreds of activities to as few as two activities. GPs’ mixed views of their CP were reported,^18^ highlighting varying degrees of GP–CP joint-working. Some GPs perceived that recently qualified pharmacists may add little value.

This study also explored instances where GP–CP joint-working was unsuccessful, identifying a lack of (a) trust between GPs and CPs in each other’s roles and skills, (b) understanding of how CPs’ knowledge and skills could be used in general practice, and (c) CP training to do practice work. These findings are supported by a recent study of practice pharmacists that showed that while pharmacists made positive contributions to practice work (for example reducing workloads and medicines waste, and increasing patient safety and experience), this was only possible after trust was developed between pharmacists and the practice team, and pharmacists understood the nature and processes of practice work.^27^ These findings notwithstanding, the present study also found that in a large number of practices, CP integration and GP–CP joint-working were successful, with CPs perceiving their experience and contribution to practice work as positive, and SCs acknowledging the value of their work. In these successful practices, CPs were accepted to the extent that they worked beyond a 'backroom' role and were trusted by GPs to work independently, including conducting patient-facing activities (for example medicines reviews in the practice and in patients’ homes). Despite the limitations discussed earlier, the positive response from patients in this study corroborate the reported success of GP–CP collaboration in many practices. The positive responses also align with findings from a recent survey where patients and carers reported being fully involved in discussions about their medicines, and feeling that they were listened to and that their concerns were addressed when they were seen by a pharmacist at their practice. The same study also found that seeing a pharmacist gave patients quicker access to an appointment at their practice and the patients did not feel the need to see their GP afterwards.^28^ It is therefore possible that not only GPs’ views of, and trust in, CPs’ knowledge, experience, and skills, but also CPs’ own initiative, confidence, and autonomy may have contributed to whether or not CP integration and GP–CP joint-working were successful in the PCPP scheme.

### Implications for research and practice

At a time where there is a strong policy drive in the UK to rapidly expand the inclusion of pharmacists in general practice,^6,7,24^ CPs are well placed to contribute. They are recognised as medicine generalists and already provide patient-facing medicines review services.^3^ The PCPP indicated the value of local CP involvement through joining up of the patient’s medicines pathway and reducing the risk of fragmented care. This model enabled CPs to continue to practice in their community pharmacy to maximise these benefits. However, the needs of practices may exceed the model that was fundable in the PCPP scheme of one clinical session per week. Hence, future initiatives can learn from this model.

Benefits of CP–GP joint-working were particularly evidenced in cases where medicines-related tasks were seen as complex by GPs, difficult to complete in a timely way, or not prioritised because of competing workloads. CPs’ medicines-focused skills and knowledge meant that these tasks were not viewed by them as complex, highlighting existing scope for role substitution between GPs and practice-based pharmacists, to enable better use of available GP time and more timely completion. Although the focus of this study on CPs', CPEs', and SCs' perspectives meant that it was not possible to ascertain whether CPs’ work created further risks to patient safety, findings from the study suggest that CPs’ work reduced the risk of discrepancies and errors otherwise known to arise, some of which had been present in patients’ records for months or years.

Evidence from this study suggests that, because tasks performed by CPs were not perceived as complex by CPs, both they and GPs may have underplayed pharmacists’ clinical contribution by sometimes seeing these activities as akin to administrative tasks. Ensuring a successful collaboration, therefore, requires that both practice teams and pharmacists understand each other’s roles. Hence, raising awareness of CPs' knowledge and skills will promote mutual trust in each other’s skills and collaborative working, with training being one way to accomplish this trust.

A recent study of the contribution of CP independent prescribers in general practice appears to corroborate this hypothesis. According to this report, CP independent prescribers, like CPs in the present study, largely contributed to the delivery of care by providing comprehensive medicines reviews with, and advice to, patients; increasing medicines adherence; and reducing the time patients needed to wait for an appointment.^29^ However, in order for a collaborative pharmacist–GP synergy to be effective, prior training proved to be required to build relationships, integrate the role of pharmacists in general practices, create a sense of belonging to the team, and develop trust in each other’s knowledge, skills, and mutual contribution.^29^

During a period of rapid expansion of primary care pharmacy, and in recognition that pharmacists can bridge the gap between general practice and community pharmacy,^8^ this study has shown the potential and actual contribution that local CPs can make to general practice work, ensuring patients receive continued and more comprehensive support throughout their healthcare journey in primary care. The small amount of time based in the general practice, mixed levels of trust between CPs and the practice team, and lack of initial training in the practice work may have limited that contribution. The PCPP scheme has shown that it is possible to integrate CPs in general practices at scale, which has strengthened GP–CP collaborations. The success of future schemes will depend on what they can learn from the PCPP with regards aligning funding and contracting, as well as training, to ensure CPs can be more effectively and quickly integrated into the practice team.
